# Data Report: Golf-Related Engagement During COVID-19 Quarantine Restrictions (4–12th May 2020)

**DOI:** 10.3389/fspor.2020.00112

**Published:** 2020-09-02

**Authors:** Graeme G. Sorbie, Alexander J. Beaumont, Ashley K. Richardson, Jonathan Glen, Scott M. Hardie, David Lavallee

**Affiliations:** ^1^Division of Sport and Exercise Sciences, School of Applied Sciences, Abertay University, Dundee, United Kingdom; ^2^School of Science, Technology and Health, York St John University, York, United Kingdom

**Keywords:** coronavirus, exercise, golf, pandemic, physical activity, SARS-CoV-2, sport

## Introduction

As a result of the COVID-19 pandemic caused by SARS-CoV-2 coronavirus, Governments around the world aimed to suppress the spread of the disease by applying various forms of quarantine (stay at home) restrictions. For example, according to the regulations set by the United Kingdom (UK) Government on 23rd March 2020, individuals should only leave their home to; shop for basic necessities, any medical need, travelling to and from essential work, and to perform one form of exercise per day (United Kingdom Government, 2020a). In particular, exercising was restricted to individuals being alone or with members of their household (United Kingdom Government, 2020a). These quarantine restrictions created unprecedented disruption to individuals' social, family, and work lives.

As a consequence of these restrictions being put in place, all sports events and recreational activities such as soccer, tennis, bowls, and golf were suspended (Parnell et al., 2020; United Kingdom Government, 2020a). With these quarantine restrictions in place, there is a significant risk emerging that concerns the potential decline in physical activity and sports-related activities, which in turn can have a detrimental effect on physical and mental health (Chekroud et al., 2018). It is well-publicised that there are various health and well-being benefits resulting from the participation in physical activity and sports-related activities (Bo Andersen et al., 2000; Saxena et al., 2005; O'Donovan et al., 2010) and, in turn, there are potential health and well-being implications as a result of the quarantine restrictions (Ricci et al., 2020). Specifically, these restrictions could be detrimental to many physiological and psychological risk factors such as coronary heart disease, obesity, stress, social isolation, negative emotions, and sleep quality (Ellingson et al., 2018; Lippi et al., 2020a,b). During the quarantine restrictions, it has been recommended that individuals partake in home-based physical activities such as aerobic exercise using a stationary bike, bodyweight strength training, dance-based exercise, and active gaming in order to counteract the negative physical and mental side effects of the pandemic (Hammami et al., 2020). With sports and recreational activities suspended, the previously outlined physiological and psychological risk factors may be increased for individuals who habitually practiced sports activities prior to restrictions (Lippi et al., 2020a).

Unlike many other sports, golf provides opportunities for individuals to complete golf-related activities within their home environment. For example, with limited golf equipment (e.g., golf clubs and balls), many golfers are able to practice full golf swings and full golf shots, as well as chipping and putting skills, all of which contribute to a large proportion of the game of golf (Keogh and Hume, 2012). Active gaming at home can also provide golfers with opportunities to practice golf-related activities. This active gaming during the quarantine restrictions has been previously recommended and endorsed by Hammami et al. (2020). There are many physical and mental health benefits of completing these golf-related activities including balance control (Sell et al., 2007; Tsang and Hui-chan, 2010; Gao et al., 2011), muscle function (Martinez Bustelo et al., 2016), and strength and flexibility (Sell et al., 2007). In addition, performing these skills during the quarantine restrictions may also provide opportunities to reduce sedentary time, which has during normal living, previously been associated with reduced cardiovascular risk factors (Young et al., 2016) and improvements in mood, stress, and sleep quality (Ellingson et al., 2018). It has been recommended that sedentary time for adults should be <7.5 h per day (Ku et al., 2018). In addition to physical golf activities, the use of technology also provides opportunities for individuals to engage in other activities such as listening to golf-related audio files, watching golf tournaments on television, and/or receiving coaching sessions online. These golf-related activities can provide individuals with a continued sense of belonging in relation to their sporting community, which can therefore provide potential well-being benefits that have been previously linked to the sport (Belanger et al., 2013; Murray et al., 2017; Sorbie et al., 2020).

As a result of restrictions to suppress the spread of the COVID-19 pandemic, and the unique opportunities that golf provides, this data report presents demographical details and golf-related activities that were performed during a period of quarantine (4–12th May 2020). In turn, these data can be used to compare golf-related activities across the different demographics.

## Methods

Data collection commenced after the project received Institutional Ethical approval, at which point the survey was published online between the 4 and 12th of May 2020. The survey was closed on the 12th May 2020 as a result of many golf courses in the UK re-opening on the 13th May 2020 (United Kingdom Government, 2020b). At the onset of data collection, the UK population had been under consistent movement restrictions for 42 days (6 weeks). The instructions for participants completing the survey were as follows: “You are being invited to participate in a 2-min survey assessing your golf activities during the Covid-19 lockdown (restricted movement to reduce day-to-day contact with other people) period. In order to be eligible for the study you must consider yourself as a social, amateur or professional golfer who participates in golf for recreational or competitive purposes. You must also be aged 16 or over. By completing this survey, you are providing consent for your anonymous data to be stored for a period of 10 years within a secured server and be used for research purposes. Participation is completely voluntary and you can withdraw from the study at any point without giving any reason.” All golfers provided informed consent before completing the survey. Following this, golfers were provided with a set of questions relating to their demographics and golf experience: age, gender, country of residence, occupation, working status (during the period of quarantine), golfer status (professional golfer, golfer holding a handicap index, and social golfer), golf handicap index (if applicable), if they were playing on outdoor golf courses (during the period of quarantine; and if so, for how long), and how many rounds of golf they completed during 2019. Secondly, golfers were provided with a set of questions relating to golf-related activities during the period of quarantine: number of times physical golf skills outdoors (e.g., golf shots and chipping into a golf net) were performed; number of times physical golf skills indoors (e.g., chipping and putting) were performed; number of times physical conditioning exercises related to golf sessions (e.g., strength and conditioning programmes, gym programmes related to golf) were performed; number of times physical virtual reality golf skills (e.g., virtual reality golf games) were performed; number of times golf-related games (e.g., golf games on a console or mobile) were performed; number of times golf was viewed on television or any device (e.g., repeats of tournaments, golf shows/movies); number of times online golf tutorials (e.g., engaging with coaching videos on social media) were viewed; number of times golf related podcasts, audiobooks, or radio shows were listened to; number of times golf-related reading materials (e.g., magazines, books, journals) were engaged with; and any other golf-related activities that were performed during the period of quarantine. For the golf-related activity questions, golfers were provided with a 5-item response scale; 0 times, 1–9 times, 10–19 times, 20–29 times, and 30+ times (see https://data.mendeley.com/datasets/pnvv34cm37/draft?a=d80312a3-8588-413b-8eb1-473f007679b2).

## Dataset

The dataset includes 1,273 golfers [the descriptive data below is reported as percentage (%) or mean ± standard deviation]. Within the dataset, 87% of the golfers are male and 13% are female, aged 53 ± 16 years. 95.2% of the golfers reside within the UK, 2% in the United States of America, 1.2% in Ireland, 0.5% in Canada, 0.3% in Australia, 0.2% in Gibraltar, 0.1% in Germany, 0.1% in France, 0.1% in the United Arab Emirates, and 0.3% did not answer. As a result of COVID-19, 19% of the golfers are working, 27% of golfers are working from home, 32% of the golfers are not working and 22% of the golfers are retired. In terms of golfer status, 93% hold a golf handicap index (15 ± 8 golf handicap index), 5% are social golfers, and 2% are professional golfers. At the time of the data collection, 33 golfers were playing golf on outdoor courses. During 2019, the golfers completed a total of 70 ± 52 rounds of golf.

Table 1 reports the number of times golf-related activities were engaged in during quarantine restrictions (4–12th May 2020) by country of residence, golfer status, gender, and working status as a result of COVID-19. Total and stratified sample sizes are indicated.

**Table 1:** The number of times golf-related activities were engaged in during COVID-19 quarantine restrictions (4–12 May 2020) by country of residence, golfer status, gender, and working status as a result of COVID-19.

		**Category**	**Outdoors**	**Indoors**	**Conditioning**	**VR**	**Games**	**TV/Device**	**Online**	**Podcasts**	**Reading**
Country (*N* = 1,269)	Australia (*n =* 4)	0	3	3	2	4	4	1	2	3	2
		1–9	1	0	1	0	0	2	1	0	2
		10–19	0	0	1	0	0	0	0	1	0
		20–29	0	0	0	0	0	1	1	0	0
		30+	0	1	0	0	0	0	0	0	0
	Canada (*n =* 6)	0	3	1	2	4	3	0	2	2	1
		1–9	1	3	4	2	2	4	3	4	3
		10–19	0	1	0	0	0	1	1	0	0
		20–29	1	0	0	0	1	0	0	0	1
		30+	1	1	0	0	0	1	0	0	1
	France (*n =* 1)	0	0	0	0	0	0	0	0	0	0
		1–9	0	0	0	0	0	1	0	0	1
		10–19	1	0	1	0	0	0	0	0	0
		20–29	0	1	0	1	1	0	0	0	0
		30+	0	0	0	0	0	0	1	1	0
	Germany (*n =* 1)	0	0	1	1	1	1	0	0	0	1
		1–9	1	0	0	0	0	1	1	1	0
		10–19	0	0	0	0	0	0	0	0	0
		20–29	0	0	0	0	0	0	0	0	0
		30+	0	0	0	0	0	0	0	0	0
	Gibraltar (*n =* 2)	0	2	2	2	2	2	1	2	2	2
		1–9	0	0	0	0	0	1	0	0	0
		10–19	0	0	0	0	0	0	0	0	0
		20–29	0	0	0	0	0	0	0	0	0
		30+	0	0	0	0	0	0	0	0	0
	Ireland (*n =* 15)	0	5	12	8	15	15	5	5	10	9
		1–9	5	1	4	0	0	4	6	3	5
		10–19	0	1	1	0	0	2	2	2	0
		20–29	1	0	1	0	0	1	0	0	0
		30+	4	1	1	0	0	3	1	0	1
	United Arab Emirates (*n =* 1)	0	1	1	1	1	1	0	1	1	0
		1–9	0	0	0	0	0	1	0	0	1
		10–19	0	0	0	0	0	0	0	0	0
		20–29	0	0	0	0	0	0	0	0	0
		30+	0	0	0	0	0	0	0	0	0
	United Kingdom (*n =* 1,214)	0	536	730	659	1,115	978	352	423	947	603
		1–9	343	242	202	55	111	446	430	196	477
		10–19	121	95	118	16	61	229	167	38	65
		20–29	45	25	75	5	15	70	59	13	29
		30+	166	118	158	17	48	114	131	18	37
	United States of America (*n =* 26)	0	4	9	7	21	19	8	8	11	4
		1–9	8	8	7	4	4	11	8	7	14
		10–19	5	3	3	1	0	3	5	6	6
		20–29	2	3	5	0	1	0	2	1	0
		30+	7	3	3	0	2	4	3	1	2
Golfer status (*N* = 1,267)	Professional (*n =* 22)	0	6	6	5	21	16	4	4	9	7
		1–9	6	6	1	1	3	7	9	9	8
		10–19	4	3	7	0	3	9	3	1	5
		20–29	0	1	1	0	0	0	2	2	0
		30+	6	6	8	0	0	3	4	1	2
	Golfer with handicap index (*n =* 1,186)	0	516	712	631	1,089	963	340	415	915	569
		1–9	334	234	212	55	105	440	420	194	480
		10–19	118	94	118	14	52	216	165	45	66
		20–29	47	28	77	6	18	71	57	12	30
		30+	169	116	147	17	48	117	126	19	39
	Social golfer (*n =* 59)	0	32	40	44	51	42	21	24	50	44
		1–9	17	13	4	5	9	27	19	8	15
		10–19	6	3	3	3	6	8	6	1	0
		20–29	1	0	1	0	0	1	3	0	0
		30+	3	2	7	0	2	2	7	0	0
Gender (*N* = 1,269)	Female (*n =* 160)	0	85	112	94	148	148	81	87	137	84
		1–9	44	31	20	9	9	58	55	18	67
		10–19	12	8	17	2	3	14	10	4	6
		20–29	6	5	8	0	0	5	3	0	1
		30+	12	4	21	0	0	2	5	1	3
	Male (*n =* 1,109)	0	470	649	587	1,016	877	287	358	841	538
		1–9	314	222	196	52	108	413	394	193	436
		10–19	115	92	110	15	58	220	165	43	66
		20–29	43	23	73	5	17	67	59	14	29
		30+	166	120	141	17	49	120	130	17	38
Working status as a result of COVID-19 (*N* = 1,264)	Working (*n =* 244)	0	105	143	137	214	173	57	65	182	122
		1–9	68	54	55	17	37	93	77	35	96
		10–19	29	11	24	6	21	49	49	14	11
		20–29	8	3	9	0	3	18	18	6	6
		30+	34	32	18	6	10	26	34	6	9
	Working from home (*n =* 347)	0	133	197	184	325	276	77	105	243	171
		1–9	115	69	56	13	28	137	136	79	138
		10–19	36	36	30	4	17	81	48	15	21
		20–29	15	12	28	1	9	21	21	2	4
		30+	46	33	48	2	17	30	37	8	13
	Not working (*n =* 398)	0	175	225	199	361	314	116	138	299	189
		1–9	104	78	65	20	42	142	142	78	157
		10–19	37	38	52	6	17	66	47	13	26
		20–29	14	9	25	3	5	23	18	3	12
		30+	68	46	57	8	20	51	51	5	14
	Retired (*n =* 275)	0	137	193	158	259	256	114	132	248	134
		1–9	71	50	40	11	10	100	92	18	113
		10–19	26	14	21	1	6	37	31	5	13
		20–29	12	5	18	2	1	10	5	3	8
		30+	29	13	38	1	2	14	15	1	5

*Category: Number of times golf-related activities were engaged in; Outdoors: During the lockdown period, how many times have you performed physical golf skills outdoors (e.g., golf shots and chipping into a net)?; Indoors: During the lockdown period, how many times have you performed physical golf skills indoors (e.g., chipping and putting)?; Conditioning: During the lockdown period, how many times have you performed physical conditioning exercises related to golf sessions (e.g., strength and conditioning programmes, gym programmes related to golf)?; VR (VR, virtual reality): During the lockdown period, how many times have you performed physical virtual reality golf skills (e.g., Wii, VR golf games)?; During the lockdown period, how many times have you performed golf related games (e.g., golf games on a PlayStation, Xbox, Switch, iPad or mobile)?; TV/Device: During the lockdown period, how many times have you watched golf on TV or any device (e.g., repeats of tournaments, golf shows/movies)?; Online: During the lockdown period, how many times have you watched online golf tutorials (e.g., engaging with coaching videos on Twitter, Facebook, YouTube)?; Podcasts: During the lockdown period, how many times have you listened to golf related podcasts, audiobooks or radio shows?; Reading: During the lockdown period, how many times have you engaged with golf related reading materials (e.g., magazines, books, journals)*.

### Strengths and Limitations

The present dataset has several strengths: it was conducted with 1,273 golfers over an 8 day period; the dataset is representative to the numbers of registered golfers, including age (Sorbie et al., 2020), gender (Lange, 2019), and golf handicap index (Golf Care, 2016); the dataset provides insights into what golf-related activities were performed during an unprecedented period of restricted movement; within the dataset participant demographics were collected, which may be useful for further analysis, replication, and extensions of the current research (i.e., age, gender, country of residence, occupation, working status at the time of data collection, golfer status, golf handicap index, golf-related activities, and completion date/time); and the data were collected during a narrow time period (4–12th May 2020) and after a prolonged period of quarantine, which reflected similar conditions for the majority of the dataset [following the closure of the survey (12th May 2020) many golf courses in the UK were permitted to reopen (United Kingdom Government, 2020b)].

The present dataset has some limitations: it only relates to the sport of golf; the dataset only presents golf-related activities that were performed during a period of quarantine restrictions (4–12th May 2020); and the dataset is only representative across limited countries and golfer status (i.e., a high percentage of the golfers are from the UK and are golfers holding a golf handicap index).

### Possible Research Paths

The dataset provides insights into the golf-related activities that were performed, and the frequencies in which they were performed, during a period of quarantine restrictions (4–12th May 2020). The dataset can be filtered according to age, gender, country of residence, occupation, working status at the time of data collection, golfer status, golf handicap index, golf-related activities, and completion date/time. Following this, the dataset can be used to compare golf-related activities across the different demographics and other data included within the dataset. For example, the dataset can provide insight into differences in golf-related activities between multiple occupations, specific working status' during COVID-19, or different golfer status'. This could enable researchers to gain an understanding of golfers' habits dependant on their status during a period of quarantine (4–12th May 2020).

In future research, the present dataset could also be used to provide recommendations if there were to be another move toward quarantine restrictions during the current pandemic, or even future pandemics. Furthermore, the dataset could be used to compare golf-related activities with other datasets (e.g., tennis-related activities) collected during quarantine restrictions. The dataset could also be used for future reports that provide insight into physical and sport-related activities that were being conducted during the quarantine restrictions.

## Dataset Description

The data in the present report have been deposited in the Mendeley repository and are freely accessible through the following link: https://data.mendeley.com/datasets/pnvv34cm37/draft?a=d80312a3-8588-413b-8eb1-473f007679b2 under the name: “Golf-related engagement during COVID-19 quarantine restrictions, 4–12th May 2020.” The dataset has been stored in a Microsoft Excel (Version 2016) format, has been anonymised throughout and any identifiers have been removed.

## Data Availability Statement

The datasets presented in this study can be found in online repositories. The name of the repository and accession number can be found in the article.

## Ethics Statement

The studies involving human participants were reviewed and approved by Abertay University. The patients/participants provided their written informed consent to participate in this study.

## Author Contributions

All authors listed have made a substantial, direct and intellectual contribution to the work, and approved it for publication.

## Conflict of Interest

The authors declare that the research was conducted in the absence of any commercial or financial relationships that could be construed as a potential conflict of interest.
